# *Ponciri Fructus Immaturus* ethanol extract attenuates septic shock through inhibition of the STAT1 signaling pathway

**DOI:** 10.3389/fnut.2022.988309

**Published:** 2022-09-15

**Authors:** Yo Sep Hwang, Jun-Pil Jang, Seong-Hoon Park, Aeyung Kim, Jae-Hyuk Jang, Hyang Ran Yoon, Suk Ran Yoon, Jun Hong Park, Hee Jun Cho, Hee Gu Lee

**Affiliations:** ^1^Immunotherapy Research Center, Korea Research Institute of Bioscience and Biotechnology, Daejeon, South Korea; ^2^Department of Bio-Molecular Science, KRIBB School of Bioscience, Korea University of Science and Technology (UST), Daejeon, South Korea; ^3^Chemical Biology Research Center, Korea Research Institute of Bioscience and Biotechnology, Cheongju, South Korea; ^4^Genetic and Epigenetic Toxicology Research Group, Korea Institute of Toxicology, Daejeon, South Korea; ^5^Korean Medicine Application Center, Korea Institute of Oriental Medicine, Daegu, South Korea; ^6^Herbal Medicine Resources Research Center, Korea Institute of Oriental Medicine, Naju-si, South Korea

**Keywords:** *Ponciri Fructus Immaturus*, sepsis, inflammation, pro-inflammatory cytokines, STAT1

## Abstract

Sepsis is a systemic inflammatory disease to infections and results in tissue damage and multiple organ failure. *Ponciri Fructus Immaturus* (PFI) is widely used in traditional medicine for allergic inflammation and gastrointestinal disorders. However, the effect of PFI on sepsis is still unknown. This study investigated the anti-inflammatory and antiseptic effects of PFI ethanol extract (PFIE) in LPS-stimulated J774 macrophages and mice with CLP- or LPS-induced sepsis, respectively. PFIE attenuates the LPS-induced production of the proinflammatory mediator NO by inhibiting the expression of iNOS in J774 cells. Real-time RT-PCR data and ELISA showed that the mRNA and protein levels of TNF-α, IL-1β, and IL-6 increased in LPS-stimulated J774 cells. However, this induction was significantly suppressed in PFIE pre-treated J774 cells. We also found that PFIE administration increased the survival rate of mice with LPS- and CLP-induced sepsis. Decreased serum levels of AST, ALT, and CK were observed after administration of PFIE, which was associated with reduced production of proinflammatory factors, such as NO, TNF-α, IL-1β, and IL-6. Moreover, PFIE suppressed the phosphorylation and nuclear translocation of STAT1 in LPS-stimulated J774 cells, suggesting that PFIE can inhibit LPS- and CLP-induced septic shock by suppressing the STAT1 activation. These findings provide the potential therapeutic relevance of PFIE in treating acute inflammatory disease.

## Introduction

Inflammation is one of the defense responses against infection, tissue injury, and other harmful conditions (1, 2). The inflammatory response involves multifactorial functions mediated by the activation of signaling pathways that regulate the expression of inflammatory factors in resident tissue cells and immune cells (3). Although this plays a critical role in host defense that is vital to health, an uncontrolled inflammatory response can lead to various diseases, such as cancer, heart disease, diabetes, and sepsis (4).

Sepsis is a systemic response syndrome that results in organ dysfunction and death. Despite advances in medical care, the incidence and mortality of sepsis are increasing (5). Thus, sepsis is globally an important public health issue to overcome. During the onset of sepsis, the initial immune recognition response is mediated by innate immune cells such as macrophages and neutrophils (6, 7). Macrophages produce proinflammatory cytokines, such as intereukin (IL)-1β, IL-6, and tumor necrosis factor (TNF)-α, to diminish invaded pathogens (8, 9). Nevertheless, the excessive release of inflammatory cytokines can lead to systemic inflammation and multi-organ damage (10). Nitric oxide (NO) also play a critical role in the progression of sepsis and septic shock. The release of robust amounts of NO is associated with circulatory disorders in septic shock that can lead to microvascular dysfunction and cellular toxicity (11, 12). NO is released through the inducible nitric oxide synthase (iNOS). The activation of iNOS has been implicated in the synergistic induction of various inflammatory cytokines, such as IFN, TNF, IL-1β, and IL-6 (13). Accordingly, the regulation of these inflammatory cytokines and iNOS-induced NO release is important for treating septic and endotoxic shock.

*Ponciri Fructus Immaturus* (PFI) is the unripe fruit of *Poncirus trifoliata Rafinesque* of the Rutaceae family. Traditionally, PFI extracts (PFIEs) have been used to treat inflammation, digestive ulcers, gastritis, and dysentery in Korea and China (14, 15). PFI has a variety of pharmacological properties, such as antiallergy (16), anti-apoptosis (17), anti-obesity (18), anti-inflammatory (19), and memory-enhancing effects (20). PFIE can inhibit the development of testosterone-induced benign prostatic hyperplasia through its anti-proliferative and anti-oxidant effects (21). It also suppresses the invasion and migration of B16 melanoma cells (22) and hepatocellular carcinoma cells (23). It has recently been reported that PFIE improves insulin resistance by regulating macrophage-mediated inflammation and enhances glucose and lipid metabolism in high-fat diet-induced obese mice (24). Moreover, PFIE has shown strong anti-adipogenic activity by downregulating the adipogenic markers, such as peroxisome proliferator-activated receptors proteins γ, sterol response element binding proteins-1, and CCAAT/enhancer binding proteins α (25). However, the effects of PFI on sepsis remain unknown.

In the current study, we investigated the impact of PFIE on the inflammatory responses induced by LPS in J774 macrophage cells. We further evaluated its anti-septic effect using LPS- and cecal ligation and puncture (CLP)-induced mouse models of sepsis.

## Materials and methods

### Preparation of *Ponciri Fructus Immaturus* extracts

We obtained lyophilized powder prepared from 70% ethanol extract of PFI from KOC Biotech (cat. No. KOC-70E-312; Daejeon, South Korea). PFI powder was dissolved in 10% dimethyl sulfoxide (Sigma, St. Louis, MO, United States) to a final concentration of 50 mg/mL, filtered through a 0.22 μm membrane, and stored at −20°C.

### Reagents and antibodies

LPS was obtained from Sigma. iNOS and β-actin antibodies were purchased from Santa Cruz Biotechnology (Dallas, TX, United States). phospho-IKKα/β, IKKα, phospho-IκBα, IκBα, phospho-STAT1, STAT1, phospho-STAT3, STAT3, and PARP antibodies were obtained from Cell Signaling Technology (Danvers, MA, United States).

### Cell culture and treatment

The murine macrophage cell line J774 was obtained from the American Type Culture Collection (Rockville, MD, United States) and maintained in Dulbecco’s modified Eagle’s medium (Gibco, NY, United States) containing 10% FBS (HyClone, Logan, UT, United States) and antibiotics (100 U/mL penicillin and 100 μg/mL streptomycin; Gibco-BRL). The cells were incubated in a humidified incubator with 5% CO_2_ at 37°C.

### Animal experiments

C57BL/6 mice (aged 6–10 weeks) used in animal experiments were purchased from the Korea Research Institute of Bioscience and Biotechnology (KRIBB; Cheongju, South Korea), and maintained in a controlled room [specific-pathogen-free, constant temperature (23 ± 2°C) and humidity (50 ± 10%)], 12-h light/dark cycle (lights on at 8 a.m.) at the Animal Experiment Center of KRIBB. Mice were orally administered with 10 and 20 mg/kg PFIE dissolved in phosphate-buffered saline (PBS; 100 μL/mouse) or vehicle control (PBS; 100 μL/mouse) for 7 days. After 24 h, sepsis was induced in the mice using LPS or via CLP. For LPS-induced sepsis, mice were intraperitoneally injected with LPS (10 mg/kg; in 100 μL PBS) or vehicle control (PBS; 100 μL/mouse) and were divided into the following groups (*n* = 10 per group): (1) sham; (2) LPS + PBS; (3) LPS + 10 mg/kg PFIE; and (4) LPS + 20 mg/kg PFIE groups. CLP was performed as described by Oh et al. (9). The mice were divided into the following groups (*n* = 10 per group): (1) sham; (2) CLP + PBS; (3) CLP + 10 mg/kg PFIE; and (4) CLP + 20 mg/kg PFIE groups. They were anesthetized using 500 mg/kg of avertin by intraperitoneal injection. The cecum was exteriorized with forceps, ligated, and punctured with a 23-gauge needle. The cecal contents were then emptied into the abdominal cavity. The cecum was returned to the peritoneal cavity, and the abdomen and skin were closed with 6.0 silk sutures. After the surgical procedure, 1 mL of PBS was administered subcutaneously as a resuscitation solution to prevent postoperative hypotension. Mice in the sham group were subjected to the same surgery except for cecal ligation and puncture. Mice survival rate was monitored for 7 days after CLP and LPS injection, after which no further loss of mice occurred. The KRIBB Institutional Animal Care and Use Committee approved all animal experiments (KRIBB-AEC-21075).

### Blood sample collection and analysis

Mice were orally administered with 10 and 20 mg/kg PFIE dissolved in PBS (100 μL/mouse) or vehicle control (PBS; 100 μL/mouse) for 7 days. After 24 h, mice (*n* = 5 per group) were subjected to LPS injection or CLP for 12 h. Blood samples were collected from mice via cardiac puncture after anesthesia with tribromoethanol (avertin; Sigma-Aldrich). The serum was collected by centrifugation at 2000 × *g* for 30 min and stored at −80°C for cytokine measurement.

### Cell viability

Cell viability was measured by the water-soluble tetrazolium salt (WST)-1 assay (Roche Diagnostics) following the manufacturer’s instructions. Briefly, 1 × 10^4^ J774 cells were seeded in 96-well plates overnight and then treated with various concentrations of PFIE for 24 h, 10 μL of WST-1 reagent was added to each well and incubated for 1 h. The conversion of WST-1 reagent into chromogenic formazan was assessed using a microplate reader.

### Measurement of nitric oxide production

Nitrate accumulation in the culture media or serum samples was considered an indicator of NO production. Total NO production was measured by mixing 100 μL of the culture medium or serum samples with an equal volume of Griess reagent (0.1% N-[1-naphthyl] ethylenediamine dihydrochloride and 1% sulfanilamide in 5% phosphoric acid) for 10 min. The absorbance at 562 nm was then measured using a microplate reader (Molecular Devices, San Jose, CA, United States). NO concentration was determined based on a sodium nitrite standard curve.

### Cytokine measurement

The levels of TNF-α, IL-1β, and IL-6 were measured using a mouse TNF-α DuoSet ELISA kit (DY-410; R&D Systems), mouse IL-1β DuoSet ELISA kit (DY-401; R&D Systems), and mouse IL-6 DuoSet ELISA kit. (DY-406; R&D Systems), respectively. All ELISAs were performed following the manufacturer’s instructions.

### Western blotting

The cells were solubilized in radioimmunoprecipitation assay lysis buffer with protease inhibitor cocktail (Sigma). Obtained proteins (10–30 μg) were separated using 8–15% sodium dodecyl sulfate (SDS)–polyacrylamide electrophoresis (PAGE) and then transferred to polyvinylidene difluoride (PVDF) membranes (Trans-Blot Turbo Transfer pack; Bio-Rad, Hercules, CA, United States). Subsequently, the membranes were incubated for 2 h in 5% skimmed milk/TBS-T buffer (Phosphate-buffered saline, with 5% powdered milk and 1% Triton X-100), probed with primary antibodies at 4°C overnight, and subsequently incubated for 1 h at room temperature with horseradish peroxidase-conjugated secondary antibodies. The membranes were developed with enhanced chemiluminescence reaction according to the manufacturer’s instructions.

### Quantitative real-time polymerase chain reaction analysis

Total RNA was extracted from the cultured cells using TRIzol RNA Isolation Reagents (Invitrogen, CA, United States) following the manufacturer’s instructions. Reverse transcription was performed using 10 μg of total RNA with a reverse transcription kit (Promega, WI, United States). Quantitative Real-Time PCR was performed using the QuantStudio 3 system (Thermo, Waltham, MA, United States). The expression of TNF-α, IL-1β, and IL-6 was assayed in a total volume of 20 μL containing AccuPower^®^ 2X GreenStar™ qPCR Master Mix (Bioneer, Daedeok-gu, Daejeon, South Korea) according to the manufacturer’s instructions. Relative mRNA levels were calculated using the 2^–ΔΔCt^ method. Duplicate measurements were made for each sample, and each group had at least three samples. The expression (Ct value) of GAPDH was not significantly different among all groups. The primer used were: iNOS(F), 5′-CAAGCACCTTGGAAGAGGAG-3′; iNOS(R), 5′-AAGGCCAAACACAGCATACC-3′; TNF-α(F), 5′-ACGG CATGGATCTCAAAGAC-3′; TNF-α(R), 5′-TGAGATAGCAA ATCG GCTGAC-3′; IL-1β(F), 5′-GAGTGTGGATCCCA AGCAAT-3′; IL-1β(R), 5′-CTTGTGCTCTGCT TGTGAGG-3′; IL-6(F), 5′-CTGATGCTGGTGACAACCAC-3′; IL-6(R), 5′-TCC ACGATTTCCCA GAGAAC-3′; GAPDH(F), 5′-ACCCAGAAGACTGTGGATGG-3′; and GAPDH(R), 5′-ACACATTG GGGGTAGGAACA-3′.

### Statistical analysis

Quantitative data are expressed using the mean ± standard deviation and analyzed by a 2-tailed unpaired Student *t*-test to compare the difference between groups. When the *P*-value was <0.05, the differences between groups were statistically significant.

## Results

### PFIE suppresses LPS-Induced NO production by inhibiting iNOS expression in J774 macrophages

**FIGURE 1:**
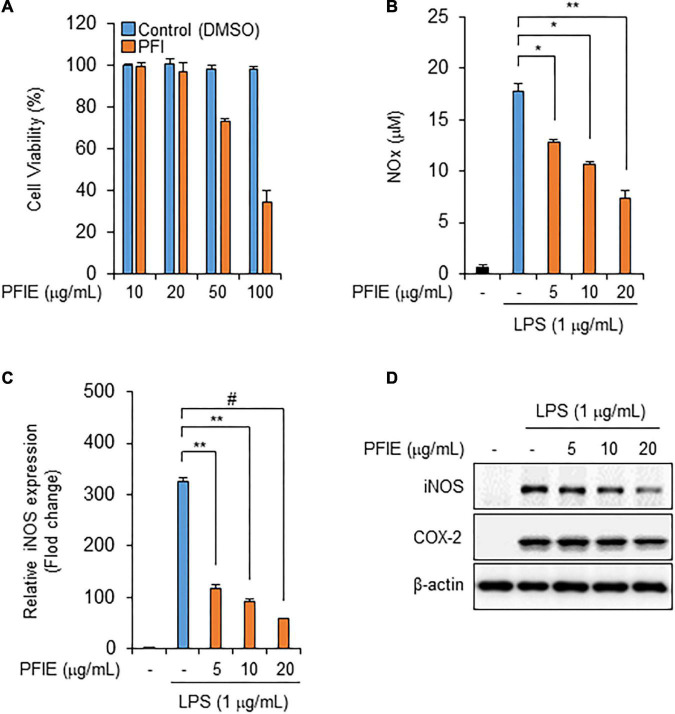
PFIE suppresses LPS-induced NO production and iNOS expression in J774 macrophages. **(A)** Effect of PFIE on J774 cell viability was determined using WST-1 assay. **(B)** NO levels in culture media were measured using Griess reagent. **(C)** mRNA levels of iNOS were analyzed using real-time PCR. Relative mRNA expression levels were normalized to those of GAPDH. *P < 0.05, **P < 0.01, and ^#^*P* < 0.001 (Student’s *t*-test). Data are representative of three experiments. **(D)** Protein levels of iNOS were analyzed via immunoblotting.

To determine the optimal concentration of PFIE, J774 macrophages were treated with varying concentrations of PFIE (10, 20, 50, and 100 μg/mL) for 24 h. PFIE decreased J774 cell viability at concentrations of 50 μg/mL but had no effect at concentrations below 20 μg/mL (Figure 1A). Therefore, the concentration of PFIE was determined below 20 μg/mL for subsequent experiments. NO is a major pro-inflammatory mediator that is overproduced in abnormal situations, thereby inducing inflammation (12). To investigate this, J774 cells were primed with the indicated concentrations of PFIE for 1 h and then treated with 1 μg/mL LPS. LPS treatment resulted in significant upregulation of NO metabolites, and this induction was significantly suppressed in PFIE-treated J774 cells in a concentration-dependent manner (Figure 1B). Furthermore, real-time PCR and western blot analysis showed that LPS induced the mRNA and protein expression of iNOS, whereas PFIE pretreatment suppressed this induction in a dose-dependent manner (Figures 1C,D). These results suggest that PFIE attenuates the LPS-induced production of the inflammatory mediator NO by inhibiting the expression of iNOS in J774 cells.

### PFIE suppresses the production of proinflammatory cytokines in LPS-stimulated J774 cells

**FIGURE 2:**
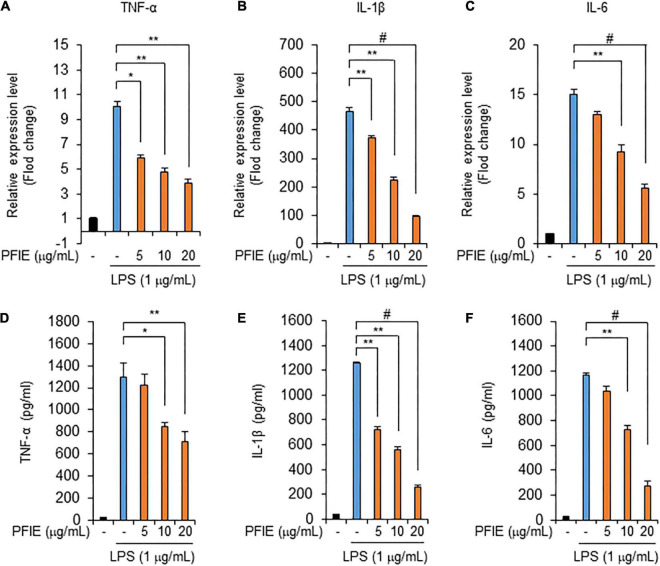
PFIE suppresses the production of inflammatory cytokines by LPS in J774 cells. Indicated PFIE concentrations were used for pretreatment 1 h prior to treatment with 1 μg/mL of LPS for 24 h. mRNA levels of panels **(A)** TNF-α, **(B)** IL-1β, and **(C)** IL-6 were determined using real-time PCR. Relative mRNA expression levels were normalized to those of GAPDH. **(D–F)** Secreted levels of panel **(D)** TNF-α, **(E)** IL-1β, and **(F)** IL-6 in the culture medium were measured using ELISA. *P < 0.05, **P < 0.01, and ^#^*P* < 0.001 (Student’s *t*-test). Data are representative of three experiments.

Next, we assessed the mRNA levels of the proinflammatory cytokines TNF-α, IL-1β, and IL-6. Real-time RT-PCR data showed that mRNA expression of TNF-α (Figure 2A), IL-1β (Figure 2B), and IL-6 (Figure 2C) increased in LPS-stimulated J774 cells, and this LPS-induced expression was attenuated by PFIE in a concentration-dependent manner. We then determined the levels of TNF-α, IL-1β, and IL-6 in the culture media using ELISA and found that these molecules were significantly upregulated in the culture media of LPS-treated J774 cells compared with that of unstimulated control J774 cells. However, PFIE significantly suppressed the production of these cytokines in a dose-dependent manner (Figures 2D–F). These data suggest that PFIE suppresses the LPS-induced production of proinflammatory cytokines in J774 cells.

### PFIE suppresses STAT1 activation mediated by LPS stimulation in J774 cells

**FIGURE 3:**
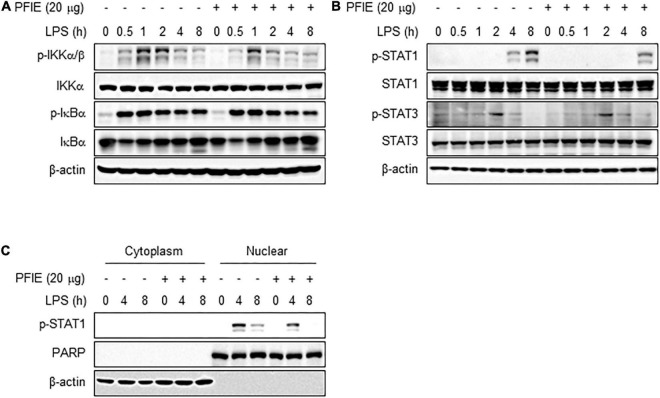
PFIE inhibits LPS-induced STAT1 activation in J774 macrophages. **(A)** IKKα/β and IκBα and **(B)** STAT1 and STAT2 phosphorylation in LPS-stimulated J774 cells in the presence or absence of PFIE was examined using immunoblotting. **(C)** pSTAT1 in the cytosol and nuclear fraction was examined using immunoblotting. PARP and β-actin were used as a nuclear and cytosolic fraction loading control, respectively.

The signaling pathway by which PFIE regulates the production of proinflammatory mediators in LPS-stimulated J774 cells is unclear. Since the LPS-induced inflammatory responses are dependent on STAT and NF-κB activity (26), we investigated the relevance of these transcription mediators on the anti-inflammatory effects of PFIE. Western blot analysis showed that there is no significant difference in the phosphorylation of IKKα/β and IκBα between LPS-treated J774 cells in the presence or absence of PFIE (Figure 3A). We next investigated whether PFIE regulated the activation of STAT1 and STAT3. STAT1 phosphorylation was observed at 4 h and was markedly increased at 8 h after LPS treatment; however, PFIE inhibited STAT1 phosphorylation in LPS-stimulated cells (Figure 3B). Meanwhile, no difference was observed in the phosphorylation of STAT3 in LPS-stimulated J774 cells with or without PFIE (Figure 3B). Moreover, LPS stimulation enhanced nuclear level of pSTAT1, which was reduced by PFIE (Figure 3C). These findings indicate that PFIE suppresses LPS-induced STAT1 activation in J774 cells.

### PFIE improves the survival rate and suppresses the inflammatory response in an LPS- and CLP-induced mouse model of sepsis

**FIGURE 4:**
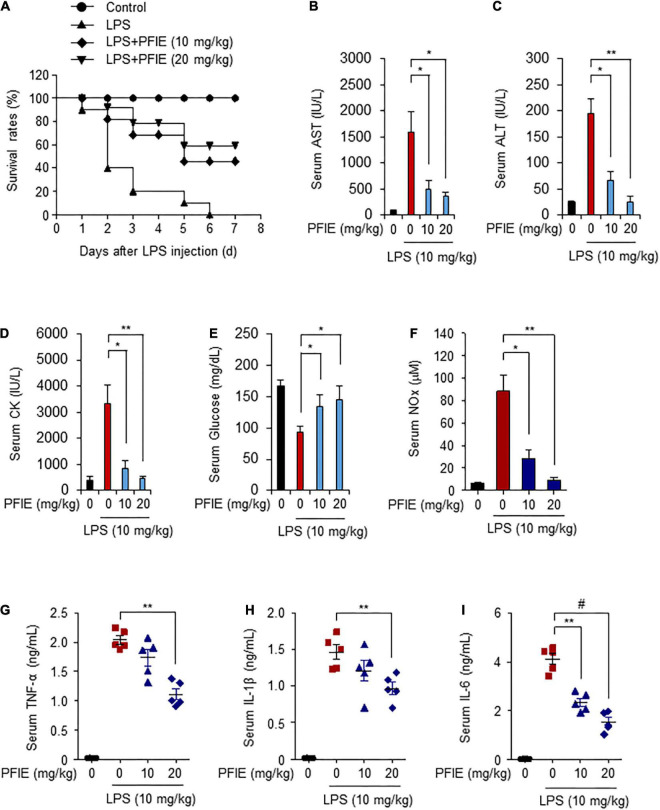
PFIE prevents septic shock and attenuates proinflammatory responses in an LPS-induced endotoxemia mouse model. **(A)** Mortality was monitored for 7 days after LPS injection in the groups (*n* = 10): PBS (circle); LPS (triangle); LPS + 10 mg/kg of PFIE (inverted triangle); and LPS + 20 mg/kg of PFIE (diamond). **(B–I)** Mouse serum samples (*n* = 5) were collected 12 h after LPS injection. Serum levels of AST **(B)**, ALT **(C)**, and CK **(D)** measured using an automatic chemical analyzer. **(F)** Serum levels of nitric oxide (NO) metabolites measured using Griess reagent. The levels of TNF-α **(G)**, IL-1β **(H)**, and IL-6 **(I)** measured using ELISA. *P < 0.05, **P < 0.01, and ^#^*P* < 0.001 (Student’s *t*-test).

LPS is a pathogen-associated molecular pattern that causes septic shock (9, 27). Because PFIE suppresses LPS-induced inflammatory responses *in vitro*, we investigated its effect on LPS-induced sepsis. Mice were orally administered with 10 or 20 mg/kg PFIE for 7 days, and septic shock was induced via injection of 10 mg/kg LPS. The survival rates of mice were monitored for 7 days. The overall survival rate was 20% in the LPS-treated mice. In contrast, the survival rate of mice in the LPS plus 10 or 20 mg/kg PFIE-treated mice was higher (40 and 60%, respectively) than that observed in the LPS-treated mice (Figure 4A). In addition, the serum levels of AST, ALT, and CK were reduced in the PFIE-treated groups (Figures 4B–D). Meanwhile, PFIE administration restored the decreased serum glucose levels induced by LPS (Figure 4E). Next, we examined the serum level of an inflammatory mediator NO in LPS-induced septic mice. LPS treatment increased NO serum level, which was reduced by PFIE (Figure 4F). Next, we examined the serum levels of the inflammatory cytokines, including TNF-α (Figure 4G), IL-1β (Figure 4H), and IL-6 (Figure 4I). The serum levels of TNF-α, IL-1β, and IL-6 significantly increased in LPS-treated mice. In contrast, PFIE treatment markedly decreased the LPS-induced serum levels of TNF-α, IL-1β, and IL-6 in a concentration-dependent manner.

**FIGURE 5:**
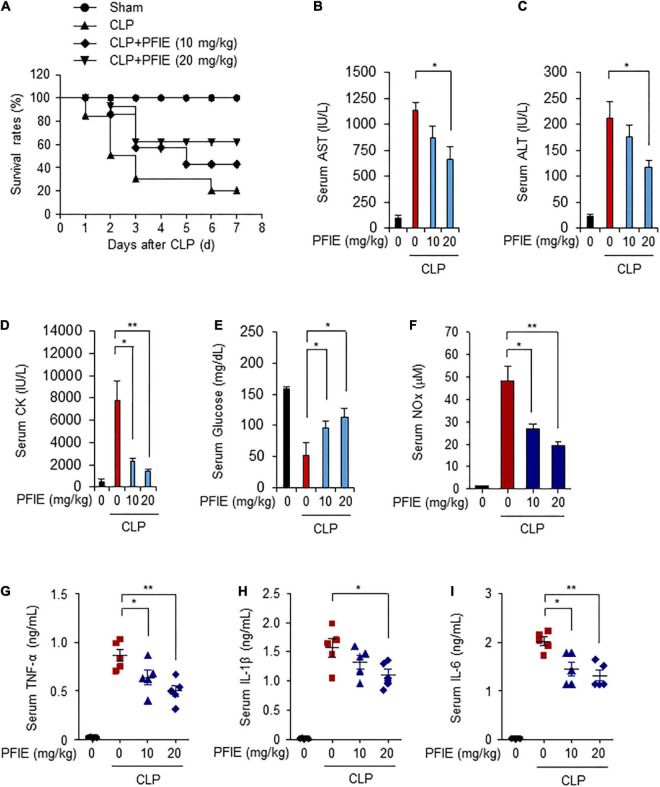
PFIE improves the survival rate and attenuates proinflammatory responses in a CLP-induced septic mouse model. **(A)** Mortality was monitored for 7 days after the surgery (*n* = 10): sham (circle); CLP (triangle); CLP + 10 mg/kg of PFIE (inverted triangle); CLP + 20 mg/kg of PFIE (diamond). **(B–I)** Mouse serum samples (*n* = 5) were collected 12 h after CLP. Serum levels of AST **(B)**, ALT**(C)**, and CK **(D)** measured using an automatic chemical analyzer. **(F)** Serum levels of nitric oxide (NO) metabolites measured using Griess reagent. Serum levels of TNF-α **(G)**, IL-1β **(H)**, and IL-6 **(I)** measured using ELISA. *P < 0.05 and **P < 0.01 (Student’s *t*-test).

We further investigated the effects of PFIE administration on the survival of septic mice using a CLP-induced septic mice model. Mice were orally administered with 10 or 20 mg/kg PFIE for 7 days and were then subjected to CLP. The survival rates of mice were monitored for 7 days. Similar to that observed with the LPS-induced model, the survival rates of PFIE-treated groups were significantly higher than those of the CLP-induced septic mice, and this increase occurred in a concentration-dependent manner (Figure 5A). PFIE administration decreased AST, ALT, and CK serum levels, which were enhanced by CLP (Figures 5B–D). Moreover, the serum levels of glucose were partially restored in the PFIE-treated groups compared with those in the CLP-only group (Figure 5E). We next assessed the effect of PFIE on the production of inflammatory factors in CLP-induced septic mice. PFIE administration markedly reduced the serum levels of NO (Figure 5F), TNF-α (Figure 5G), IL-1β (Figure 5H), and IL-6 (Figure 5I) in CLP-induced septic mice. These findings indicate that PFIE enhances the survival of LPS- and CLP-induced septic mice by suppressing inflammatory responses.

## Discussion

Inflammation is a complex biological response to noxious stimuli, such as foreign pathogens or tissue damage (1). The inflammatory response is important for host defenses against infection. However, abnormal activation of innate immune cells can lead to sepsis, a systemic response syndrome resulting in organ dysfunction and death (28). Despite advances in medical care, the incidence and mortality of sepsis are increasing. Thus, sepsis is a severe public health problem to overcome worldwide. Therefore, it is necessary to develop efficient antiseptic therapeutics for patients with sepsis. PFI has various pharmacological properties such as antiallergy, antitumor, and anti-inflammatory effects. Although many studies have investigated the pharmacological effects of PFIE on various diseases, its effects on the underlying mechanisms of the pathogenesis of sepsis are unclear. Here, we demonstrated the antiseptic effect of PFIE *in vivo* using LPS- and CLP-induced mouse models of sepsis. PFIE administration improved the survival rate of LPS- and CLP-induced septic mice. AST, ALT, and CK serum levels are typically elevated in inflammatory shock conditions, and their high serum levels may be a sign of liver injury. Also, an imbalance of glucose in the blood and tissues is one of the hallmarks of sepsis. Therefore, controlling blood glucose levels is one of the vital treatments for sepsis (5, 29). Our data showed that the serum levels of ALT, AST, and CK were significantly reduced by PFIE treatment in LPS- and CLP-induced septic mice, which indicates that it suppressed endotoxin- and CLP-induced liver damage. Moreover, the serum level of glucose decreased by LPS- and CLP-induced septic shock was returned to normal levels by PFIE. These findings indicate the therapeutic potential of PFIE for sepsis.

Sepsis is associated with excessive activation of the innate immune system. Macrophages and neutrophils play critical roles in regulating cytokine-mediated inflammatory responses against infection (6). Macrophage-mediated proinflammatory responses to infection generally allow pathogen clearance with minimal tissue damage and remodeling. However, the uncontrolled inflammatory response of macrophages causes excessive cytokine production, which can lead to local and systemic inflammation (30). TNF-α is a critical mediator of immune response to sepsis. TNF-α levels are elevated in patients with septic shock and are associated with clinical outcomes (10). IL-1 and IL-6 are also important inflammatory cytokines in sepsis. Their excessive production is correlated with hypotension, multiorgan failure, and mortality in septic patients (8, 10). NO also is a critical mediator of the progression of sepsis. The excessive production of NO causes microvascular dysfunction and cellular toxicity (12). In the present study, we showed the anti-inflammatory effect of PFIE in endotoxin-treated macrophages and septic mouse models. PFIE suppressed the production of NO, IL-1β, TNF-α, and IL-6 in LPS-stimulated J774 cells. In addition, we extended this analysis *in vivo* using the LPS- and CLP-induced mouse model of sepsis. PFIE administration suppressed the production of NO, IL-1β, TNF-α, and IL-6 in LPS- and CLP-induced septic mice. Therefore, PFIE is predicted to suppress dysfunctional inflammatory responses by macrophages and protect against the detrimental effects of sepsis. Since the possibility that PFIE may modulate inflammatory responses by other immune cells in sepsis cannot be excluded, further research is needed to elucidate the relationship with other immune cells, such as neutrophils, in the anti-septic efficacy of PFIE.

PFI is widely used in traditional medicine for edema, dyspepsia, and cardiovascular diseases (14). PFI has various pharmacological properties such as antiallergy, antitumor, antiviral, and anti-inflammatory effects (16–20). PFI contains high amounts of various compounds, including flavonoids (naringin, neoponcirin, and poncirin) and coumarins (umbelliferone, imperatorin, and auraptene), which have various pharmacological activities, including anticancer and anti-inflammatory effects (24, 25). Naringin exhibited anti-inflammatory, antioxidant, and antiapoptotic effects in treating LPS-induced cardiac injury (31, 32). Poncirin suppressed the LPS-induced production of inflammatory cytokines TNF-α and IL-6 in RAW 264.7 macrophages and attenuated ischemic injury in stroke mice (33, 34). Auraptene suppressed inflammatory responses by inhibiting LPS-induced p38 activation in RAW 264.7 macrophages and ameliorated neuronal cell death in the LPS-induced Parkinson’s disease mouse model (35, 36). Umbelliferone prevented testicular injury by suppressing oxidative damage and inflammation in diabetic rats (37). Imperatorin mitigated cardiac dysfunction by modulating inflammation and oxidative stress in high-fat diet rats (38). It also attenuated the inflammation associated with synovial fibrosis by suppressing the production of proinflammatory cytokines and mediators (39). Therefore, the anti-inflammatory properties of PFIE during sepsis are based on various pharmacological activities.

NF-κB and STAT1 are vital in inflammatory and immune responses (40). NF-κB signaling pathway is activated in peripheral mononuclear cells and alveolar macrophages of sepsis patients, which is highly correlated with the progression of sepsis (41). STAT1 is also a critical transcription factor involved in many physiological functions, including the response to infection. STAT1 activation is required to produce interferons, which trigger the JAK/STAT signaling pathway in an autocrine or paracrine manner. In septic mouse models, STAT1 activity is significantly elevated in macrophages (42, 43). Although several flavonoids inhibit the production of proinflammatory cytokines by suppressing NF-κB activity (44). Oroxylin A, a flavonoid isolated from Scutellaria baicalensi, attenuates the expression of proinflammatory cytokines by suppressing the JAK/STAT1 pathway without affecting NF-κB activity (45). Consistent with this result, we showed that PFIE suppressed the activation of STAT1, but not NF-κB, and sequentially reduced the LPS-induced production of inflammatory cytokines in J774 cells. These findings suggest that the anti-inflammatory effects of PFIE are attributable, at least in part, to the inhibition of STAT1 activity.

## Conclusion

**FIGURE 6:**
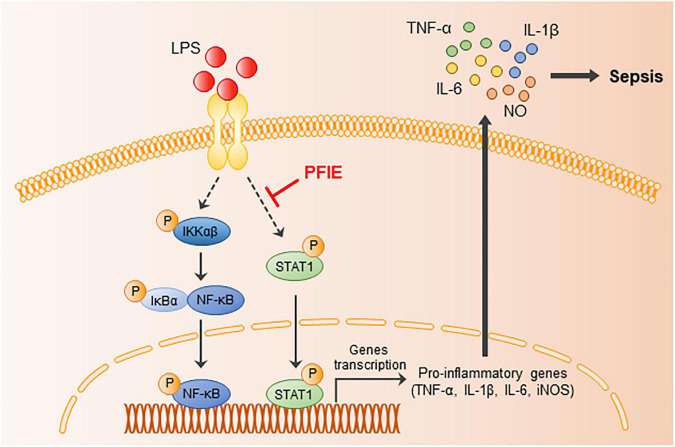
The possible inhibitory pathways of PFIE in sepsis.

In summary, we revealed for the first time the anti-septic potential of PFI extract and its molecular signaling pathway (Figure 6). The findings of this study showed that PFIE attenuated the LPS-induced inflammatory responses in J774 macrophage cells, which might be related to the inhibition of STAT1 signaling. Moreover, PFIE administration ameliorated experimental sepsis in mice by suppressing the production of inflammatory cytokines and mediators. This study demonstrates the role of PFIE as a promising therapeutic candidate in treating acute inflammatory disease.

## Data availability statement

The original contributions presented in this study are included in the article/supplementary material. Further inquiries can be directed to the corresponding authors.

## Ethics statement

The animal study was reviewed and approved by the KRIBB Institutional Animal Care and Use Committee.

## Author contributions

YH and J-PJ: conceptualization. YH, J-PJ, AK, and HY: methodology. J-HJ: formal analysis. YH, J-PJ, HY, and SY: investigation. JP and S-HP: data curation. YH and JP: writing—original draft preparation. JP, HC, and HL: writing—review and editing and supervision. HC and HL: project administration. All authors contributed to the article and approved the submitted version.
